# Time restricted feeding modifies leukocyte responsiveness and improves inflammation outcome

**DOI:** 10.3389/fimmu.2022.924541

**Published:** 2022-11-02

**Authors:** Krisztina Ella, Ágnes R. Sűdy, Zsófia Búr, Bence Koós, Ármin S. Kisiczki, Attila Mócsai, Krisztina Káldi

**Affiliations:** Department of Physiology, Semmelweis University, Budapest, Hungary

**Keywords:** adipocyte, arthritis, circadian, metabolic rhythm, neutrophil, inflammation, leptin

## Abstract

Time restricted eating, the dietary approach limiting food intake to a maximal 10-hour period of daytime is considered beneficial in metabolic dysfunctions, such as obesity and diabetes. Rhythm of food intake and parallel changes in serum nutrient levels are also important entrainment signals for the circadian clock, particularly in tissues involved in metabolic regulation. As both the metabolic state and the circadian clock have large impact on immune functions, we investigated in mice whether time restricted feeding (TRF) affects systemic inflammatory potential. TRF slackened the symptoms in K/BxN serum-transfer arthritis, an experimental model of human autoimmune joint inflammation. Compared to *ad libitum* conditions TRF reduced the expression of inflammatory mediators in visceral adipose tissue, an integrator and coordinator of metabolic and inflammatory processes. Furthermore, TRF strengthened the oscillation of peripheral leukocyte counts and alongside decreased the pool of both marginated and tissue leukocytes. Our data suggest that the altered leukocyte distribution in TRF mice is related to the attenuated expression of adhesion molecules on the surface of neutrophils and monocytes. We propose that TRF modifies both rhythm and inflammatory potential of leukocytes which contribute to the milder reactivity of the immune system and therefore time-restricted eating could serve as an effective complementary tool in the therapy of autoinflammatory processes.

## Introduction

The circadian clock is an endogenous time-measuring system which controls the daily rhythm of basic physiological processes and thereby it is a crucial factor of adaptation to oscillating environmental changes (1). External cues (called *Zeitgebers*) such as light and temperature changes or food intake can reset the phase of the circadian rhythm through the *entrainment* process (2, 3). Molecular components of the circadian clock and rhythmic molecular changes were detected in almost all tissues in mammals. The tissue clocks are organized in a hierarchical system. The central clock - located in the suprachiasmatic nuclei (SCN) - receives input from the retina and different brain regions and by integrating photic and non-photic information it synchronizes the clocks of the peripheral tissues through humoral and neuronal signals (3–5).

Operation of the molecular oscillators depends on transcriptional/translational feedback mechanisms. In the core oscillator the BMAL1/CLOCK complex induces the expression of the *Cryptochrome* (*Cry*) and the *Period* (*Per*) genes. PER and CRY form the negative factor complex, which enters the nucleus and with a certain delay inhibits the activity of the BMAL1/CLOCK complex and thereby its own expression. Following the degradation of PER and CRY, the positive element is reactivated and a new cycle begins. Through nuclear receptors, additional feedback loops can stabilize the system, as REVERBs inhibit, whereas RORs activate the transcription of BMAL1. As a result of these molecular events, most clock components show rhythmic oscillation at both the RNA and the protein levels (3–5). The BMAL1/CLOCK complex can induce the transcription of *clock controlled genes* (*ccg*) as well, which convert internal time information to rhythmic operation of different physiological processes (5, 6). In mammals more than 80% of the genes are under the control of the circadian clock at least in one tissue (7).

Disturbed circadian rhythm due to an altered molecular clock function or misalignment between the endogenous and the environmental rhythm is associated with health risks (6). Cardiovascular diseases, metabolic problems like obesity or diabetes, malignant transformations and inflammatory diseases show increased incidence in the population with circadian rhythm disturbances (6, 8–14). The prevalence of exogenous clock disruptions is increasing in our society, they affect more than half of the population for shorter or longer periods (12, 15, 16).

Circadian regulation, metabolism and the immune system form a network with pairwise interactions between the components. The bidirectional link between metabolism and the circadian clock is supported by both epidemiological and experimental data. On one hand, nutrient composition and feeding time act as efficient *Zeitgebers*, on the other hand, the circadian clock coordinates the timing of different metabolic activities (17, 18). Due to the precise temporal regulation, antagonistic pathways such as catabolic and anabolic processes are separated in time. While light is the ultimate *Zeitgeber* of the SCN, nutrient availability and feeding rhythm may constitute the primary input for the circadian clock of the liver and the adipose tissue, the central players of metabolism (19–21). As a consequence, contradictory time cues may lead to desynchrony between the central and peripheral clocks (19, 20). As an important breakthrough in the field, Panda and co-workers showed that in mice fed a high-fat diet, disruption of the normal feeding cycle by feeding in the resting (light) phase or *ad libitum* leads to obesity and the development of metabolic disorders (22). In contrast, preserving natural feeding rhythms *via* time-restricted feeding (TRF) prevented disturbance of the metabolic state without altering caloric intake (22). Also, irregular timing of food intake, which is a common practice in the modern society can profoundly perturb the human metabolic rhythm (21).

The circadian clock impacts different levels of the immune response. Disrupted clock operation is often associated with upregulation of inflammatory processes (9, 23–26). At the cellular level, the circadian clock has been shown to influence both the temporal pattern and the intensity of leukocyte responses and also the trafficking of the immune cells (4, 26–30). This can be behind the fact that the outcome of microbial infections varies depending on the time of the day when the microbial exposure occurred (31–33). Furthermore, several immune-related diseases show circadian variations in the exacerbations of symptoms; *e.g.* symptoms of allergic rhinitis, bronchial asthma and rheumatoid arthritis typically worsen during the late night and in the early morning (34–39).

Metabolism and immune functions are also tightly interconnected (40, 41). It is well known that in metabolic disorders such as metabolic syndrome and diabetes, several levels of the immune functions are negatively affected and the incidence of infections is increased (42, 43). On the other hand, inflammatory processes, such as low-grade inflammation of the visceral adipose tissue in obesity and beta cell destroying autoimmune reactions in type 1 diabetes, are important contributors of the metabolic disturbance (44). Clinical data indicate that in the treatment of autoimmune and inflammatory diseases different diets with longer fasting periods and caloric restriction attenuate the severity of symptoms (45–48). However, it is completely unknown whether food intake restricted to a well tolerable period of the day without caloric restriction and changes in the nutrient composition can affect the progression of these diseases.

As time restricted feeding (TRF) was shown to affect metabolic functions as well as the circadian system (22), and both of them control the immune system, we aimed to examine the effect of TRF on inflammatory responsiveness. We found that in mice subjected to TRF, symptoms of autoinflammatory arthritis were attenuated as compared to mice with *ad libitum* food availability. TRF strengthened rhythmic physiology and reduced inflammatory properties of the visceral adipose tissue. Moreover, after time restricted food intake, daily fluctuation in the peripheral neutrophil and monocyte counts was enhanced. Increased levels of circulatory leukocytes in the TRF group compared to the *ad libitum* (AL) group coincided with reduced margination of the neutrophils to the vessel wall and lower expression of adhesion molecules on the cell surface. Our results indicate that TRF has significant impact on inflammatory responsiveness and suggest that synchronization of food intake with the environmental and activity rhythm can function as a novel therapeutic support in the treatment of autoimmune or inflammatory diseases.

## Materials and methods

### Animals and diets

**Figure 1:**
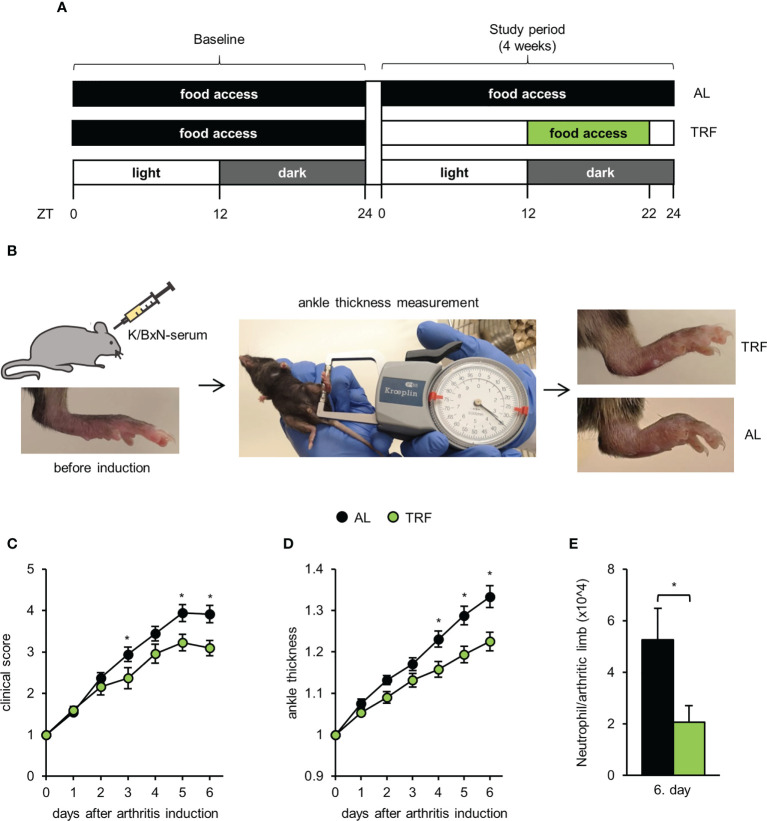
Time restricted feeding attenuates symptoms of autoinflammatory arthritis. **(A)** Schematic outline of the feeding regimens. **(B)** Short outline of the experimental setup and representative images of hind limbs of TRF and AL animals on the 6^th^ day after arthritis induction. For further details, see Materials and Methods. Mice were injected with K/BxN arthritic serum i.p. on day 0. Arthritis development was followed by clinical scoring of the limbs **(C)** and ankle-thickness measurement **(D)**. Data were normalized to values obtained on day 0 (mean ± SEM, n = 25 (AL) and n = 23 (TRF), repeated measures ANOVA, clinical score: group effect p = 0.053, group*day effect p = 0.0001, ankle-thickness: group effect p = 0.0056, group*day effect p = 0.0003, *post-hoc* Fisher test, * p < 0.05) **(E)** Neutrophil counts in the arthritic hind limbs of mice (mean + SEM, n = 7 (AL), n = 7 (TRF) mice, two-sample t-test, * p < 0.05).

Male C57BL/6 mice were bred and housed in a conventional animal facility under 12 hour light/12 hour dark schedule. *Zeitgeber* time (ZT) 0 indicates the onset of light, whereas ZT12 indicates the onset of darkness. Before the experiments mice were kept on an *ad libitum* normal chow diet and had unlimited access to water. At 60-80 days of age animals were assigned to different feeding regimens for 4 weeks in 2 groups: *ad libitum* fed (AL) and time restricted fed (TRF) groups (**Figure 1A**). TRF mice had food access in a 10 hour window in their active phase between ZT12 and ZT22 controlled by an automated FeedTime^®^ system (TSE Systems). Both groups were fed normal chow, had unlimited access to water and were kept on a grid to avoid snacking and coprophagy. Animals’ weights and food intake were measured weekly. Experiments and tests were carried out after 4 weeks. All animal experiments were approved by the Animal Experimentation Review Board of the Semmelweis University and the Government Office for Pest County (Hungary) (Ethical approval: PE/EA/1967-2/2017).

### K/BxN serum-transfer arthritis

Autoimmune arthritis was induced by a single intraperitoneal injection of 250 μl K/BxN arthritic serum. The feeding-regime was continued during the arthritis development. Serum was obtained as described previously (49). Arthritis severity was assessed daily by two investigators separately at ZT5 for 6 days by clinical scoring of the paws on a 1-10 scale including half points (49) and by measuring the ankle thickness with a spring-loaded caliper (Kroeplin). Ankle thickness values measured by the two investigators were averaged. Sum of scores of 4 limbs and mean of ankle thickness of the hind limbs were normalized to values obtained on the day of the injection (day 0). The investigators were blinded for the origin and treatment of the mice.

Six days after arthritis induction, mice were sacrificed and hind limbs were cut, minced and incubated in a digesting solution (1 ml/sample: 200 mM HEPES (pH 7.4), 200 *μ*g/ml Liberase (Roche) and 1 *μ*g/ml DNase I in HBSS) for 1 hour at 37°C 1400 rpm. To generate single cell suspensions, samples were passed through a 40 *μ*m filter and absolute cell counts were determined using CountBright™ (Invitrogen) (CytoFLEX, Beckman Coulter). Cells then were labeled with anti-Ly6G-FITC and anti-CD11b-APC. Neutrophils were identified according to their Ly6G expression and their FSC and SSC properties. For analysis of flow cytometric measurements (CytoFLEX, Beckman Coulter) the Kaluza Analysis Software (version 2.1, Beckman Coulter) was applied.

### Intraperitoneal glucose tolerance test

Mice were fasted for 16 hours before the intraperitoneal injection of glucose (1 mg/g body weight, at ZT14). Blood glucose levels were measured with a Dcont Trend glucose meter immediately before, and 1 and 2 hours after glucose administration.

### Gene-expression analysis

Visceral adipose tissue was isolated, immediately frozen in liquid nitrogen and then grinded. Adipocytes and total bone marrow isolates were immediately lysed using the TriPure^®^ Isolation Reagent (Roche). Samples were stored at -80°C until RNA preparation. Total RNA was extracted according to the manufacturer’s protocol. Following DNase treatment, cDNA was synthesized using the QuantiTect^®^ Reverse Transcription Kit (Quiagen) according to the manufacturer’s instructions. Relative expression levels of *per2*, *reverbα*, *cxcl12*, *tnfα, il1β*, *il18*, *nlrp3*, *leptin* and *adipsin* were measured in a Light Cycler^®^ 480 system (Roche) using TaqMan hydrolysis probes (see **Supplementary Table 1**). *Rplp0* was used as a reference. The second derivate maximum method was applied for data analysis using LightCycler^®^ Relative Quantification Software (version 1.5.0.39, Roche).

### Analysis of leukocyte subsets and migratory factors expressed on leukocytes

Before and after the 4 weeks conditioning, blood was collected by tail snip and absolute leukocyte number was determined. Blood collections at night (at ZT13, 17 and 21) were carried out under red light. Leukocytes (CD45+ cells) were further analyzed by flow cytometric measurements (CytoFLEX, Beckman Coulter) and different subsets were quantified. For neutrophil staining Ly6G, for identification of T- and B lymphocytes antibodies against CD3 and CD19 were applied. Monocyte staining was performed with antibodies against Ly6C, CD11b and CD115 and the population was further divided into Ly6C^high^ and Ly6C^low^ subsets as inflammatory and non-inflammatory monocytes. For data analysis the Kaluza Analysis Software (version 2.1, Beckman Coulter) was used. Cell surface expression of migratory factors of neutrophils (Ly6G+) and monocytes (CD11b+, Ly6G-, SSC^low^) were determined using antibodies listed in **Supplementary Table 2**. For gating strategies see **Supplementary Figures 1, 2**.

### Leptin treatment of leukocytes

After tail snip of non-conditioned *ad libitum* fed animals, 40 μl blood samples were collected at ZT1 and incubated in serum free RPMI 1640 medium (supplemented with 50 units/ml penicillin and 50 μg/ml streptomycin) for 30 min at 37°C, with 5% CO_2_ followed by a treatment with either 15 ng/ml leptin (Sigma-Aldrich) or vehicle for 4 hours. The applied leptin concentration (15 ng/ml) was the sum of the maximal concentration measured in the AL serum at ZT1 (10 ng/ml) and the difference between the TRF and the AL groups (5 ng/ml) at this time point. Following the treatment, expression of adhesion molecules was analyzed as described in the previous section.

### Quantification of marginated and tissue leukocytes in the lungs

Mice were anesthetized by inhalation of isoflurane and anti-CD45-PECy7 (9 *μ*g/200 *μ*l) antibody was administered by retro-orbital injection. After 5 minutes, mice were sacrificed and after a left ventricular heart puncture the lung vasculature was perfused *via* the right ventricle with 15 ml PBS and lungs were removed. To count and label pulmonary leukocytes, small lung pieces were minced in 15*μ*g/30 *μ*l anti-CD45.2-FITC, then were incubated in a digesting solution (1 ml/sample: 1% BSA, 1 mg DNase I and 0.1 mg trypsin inhibitor in PBS) for 10 minutes at 37°C. After that, collagenase XI (0.1 mg) was added and samples were incubated for an additional 20 minutes. To generate single cell suspensions, samples were passed through a 40 *μ*m filter and the absolute leukocyte counts were determined (CytoFLEX, Beckman Coulter). Cells were then labeled with live/dead-APC and anti-Ly6G-PE. Marginated (i.e. adhered to the vessel wall) (CD45-PECy7+, CD45.2-FITC+) and tissue (interstitial and alveolar) leukocytes (CD45-PECy7-, CD45.2-FITC+) were distinguished (**Supplementary Figure 3**; **Figure 7A**). Macrophages were identified according to their high autofluorescence. For analysis of flow cytometric measurements (CytoFLEX, Beckman Coulter) the Kaluza Analysis Software (version 2.1, Beckman Coulter) was applied. For gating strategies see **Supplementary Figure 3**.

### Quantification of CXCL12, leptin and corticosterone

After flushing femur bone marrow with 1 ml cold PBS, cells were spun down for 3 min at 500 g at 4°C. Supernatants were collected and stored at -80°C. CXCL12 levels were determined in the total bone marrow extracellular fluid using ELISA reagents (R&D Systems). Data were normalized to the total protein concentration measured in the supernatants using DC Protein Assay (Bio-Rad).

For serum preparation mice were anesthetized by inhalation of isoflurane and 1 ml blood was collected from the retro-orbital plexus. Samples were incubated at room temperature for 1 hour and centrifuged at 4°C and 1500 g for 10 minutes. Serum was collected and stored at -80°C. CXCL12, leptin and corticosterone levels were determined using ELISA reagents (R&D Systems).

### Statistical analysis

Statistical analysis was performed using Statistica software version 13.5 (StatSoft). Statistical significance threshold was set at p<0.05. Measurements were taken from distinct samples. Comparisons between two groups were carried out by two sample t-tests. For the analysis of parameters measured in the course of the day, one-way ANOVA and cosinor analysis were used to observe time effect. Group differences were tested by two-way ANOVA. For the analysis of IPGTT and arthritis development repeated measures ANOVA was performed, followed by Fisher’s LSD *post-hoc* test. Cosinor analysis was run in Matlab R2021a (MathWorks).

## Results

### Time restricted feeding attenuates symptoms of autoinflammatory arthritis and affects time-of-the-day-specific alterations in systemic immune activity

Mice kept under 12 hour light/12 hour dark schedule and fed normal chow were conditioned to TRF by limiting the food intake to the first 10 hours of the dark (active) phase (**Figure 1A**). Adrenal gland weight is considered an indicator of the endocrine and nervous system responses to daily or periodic stressors (50). However, gland weights in the AL and TRF groups were similar (**Supplementary Figure 4A**). As an additional marker of stress, we determined serum corticosterone levels but no significant difference between the TRF and AL samples could be obtained at either ZT1 or ZT13 (**Supplementary Figure 4B**). These data suggest that TRF did not induce serious stress in the animals. To examine the effects of TRF on the general metabolic state, body weight, food intake and blood glucose levels were followed. There was no significant difference in the animals’ body weight between the two groups either at the start or at the end of the feeding regimens (**Supplementary Figure 4C**), thus, similarly to previous findings (22), this short period of TRF did not significantly change body weight. TRF and AL mice consumed similar amount of chow (**Supplementary Figure 4D**), indicating that there was no caloric restriction in the TRF group. We also followed the food intake of the AL fed animals separately during the light and dark phases of the day. Similarly to previous data (22), food intake was not limited to the dark phase, the animals consumed 24% of the chow during the light phase (**Supplementary Figure 4E**). At the end of the 4-week feeding regimen glucose tolerance was assessed. Blood glucose levels in the two experimental groups did not differ and were in the normal range at all tested time points (**Supplementary Figure 4F**). In summary, the applied feeding program did not lead to changes in the basic metabolic parameters of the animals.

To investigate whether the applied TRF protocol can affect the development of an autoinflammatory disease, after 4 weeks of conditioning to TRF, K/BxN serum-transfer arthritis was induced. This method is considered an ideal model to study the effector mechanisms involved in the acute progression of human rheumatoid arthritis (for review see (51)). Body weight was controlled, as it can affect joint inflammation, but no significant difference between the two groups was found (**Supplementary Figure 5**). Arthritis severity was assessed daily by clinical scoring of the paws and ankle thickness measurements (**Figure 1B**). From day 4 after arthritis induction, both indicators significantly differed in the two groups of mice (**Figures 1C, D** **)**. On day 6, when inflammation severity was the most intense, in TRF animals the arthritis induced changes of clinical score and paw size displayed only 77% and 70%, respectively, compared to AL fed mice. We also investigated the cellular composition of the arthritic limbs on day 6 and found significantly reduced neutrophil count in the TRF animals (**Figure 1E**). As in this model of rheumatoid arthritis, neutrophils are the main effector cells, the lower neutrophil infiltration might significantly contribute to the attenuation of inflammation in the TRF group. These data suggest that TRF might be an effective tool in dampening the symptoms of rheumatoid arthritis.

**Figure 2:**
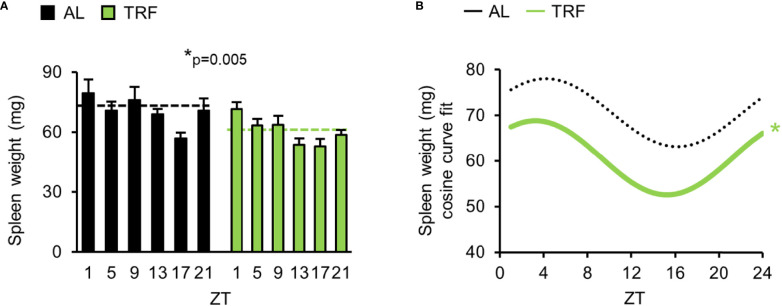
Time restricted feeding reduces spleen weight. **(A)** Spleen weight in the course of the day after the 4-week feeding regimens (mean + SEM, n = 3-11 (AL), n = 6-13 (TRF), two-way ANOVA, time effect p = 0.008, * indicates the significant group effect). Dashed lines indicate the daily average spleen weight. **(B)** Cosine curve fit to data showed in **(A)**, * and the solid line indicate significant, fixed 24-hour period cosine curve fit with cosinor analysis, whereas dashed line shows non-significant fit. Parameters of the fitted cosine curves are listed in **Supplementary Table 3**. ZT, *Zeitgeber* time.

As irregular food intake and the emerging metabolic disturbance often correlate with systemic inflammation, we hypothesized that a low-grade systemic inflammation may be triggered by AL feeding, leading to the development of a more severe form of arthritis. To search for differences between TRF and AL mice in the steady-state conditions of the immune system (corresponding to the state before arthritis induction), we measured spleen weight, which is considered an indicator of low-grade systemic inflammation (52, 53). TRF reduced the average spleen weight (**Figure 2A**), and resulted in significant rhythmicity of daily weight changes (**Figure 2B**; **Supplementary Table 3**), indicating that timely controlled food intake strengthens rhythmic physiology of the spleen and lowers systemic inflammatory state.

### TRF strengthens rhythmic functions and reduces inflammatory state of the visceral adipose tissue

**Figure 3:**
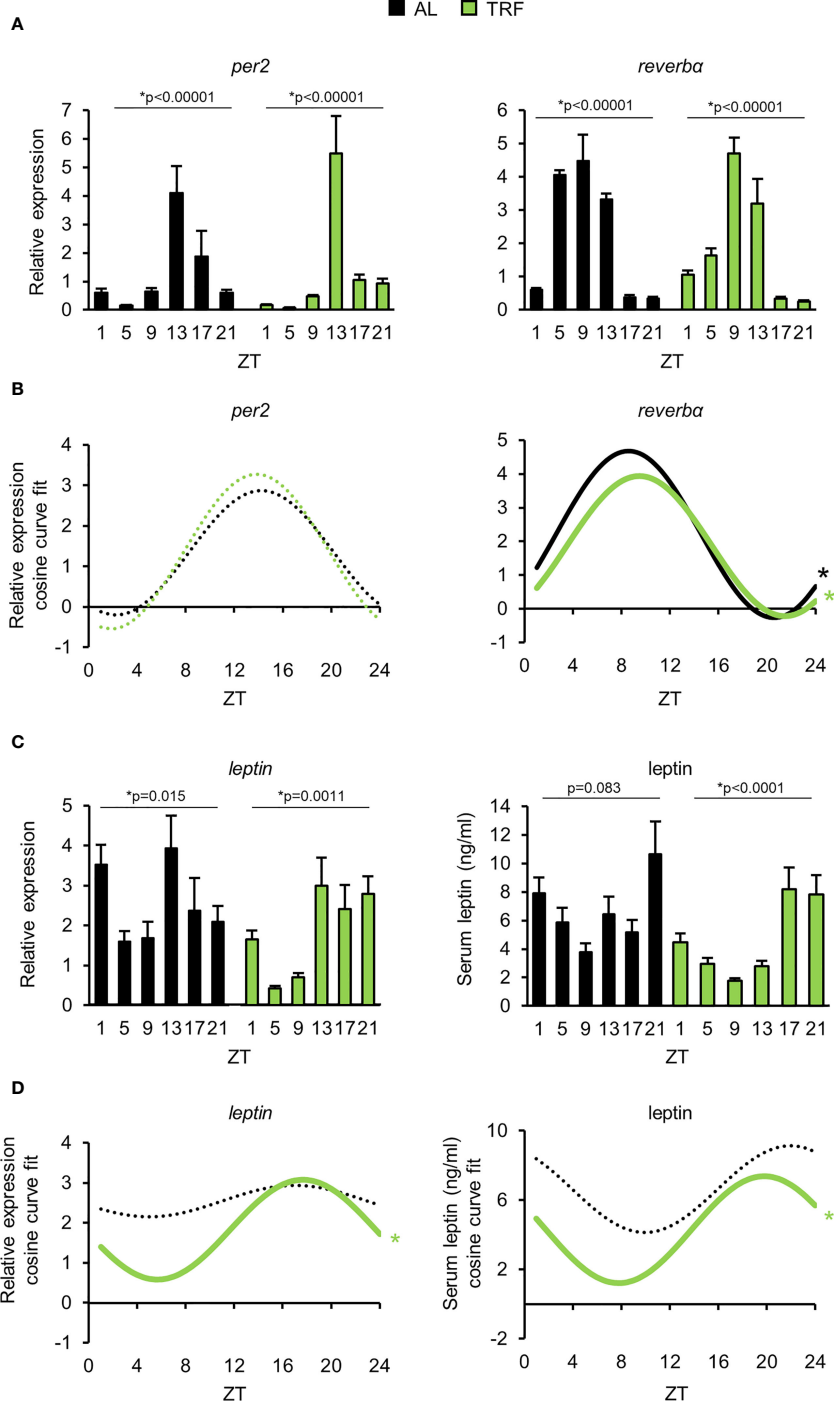
Time restricted feeding entrains the peripheral clock of visceral adipose tissue. **(A)** Relative mRNA expression of *per2* and *reverbα* in the course of the day in visceral adipose tissue (mean + SEM, n = 3-9 (AL), n = 6-11 (TRF), one-way ANOVA, * indicates significant time effect. **(B)** Cosine curve fit to relative expression data showed in **(A)**. **(C)** Relative mRNA expression of *leptin* (mean + SEM, n = 3-9 (AL), n = 6-11 (TRF)) and serum leptin levels in the course of the day (mean + SEM, n = 3-7 (AL), n = 3-8 (TRF)), one-way ANOVA, * indicates significant time effect. **(D)** Cosine curve fit to data showed in **(C)**. In mRNA expression measurements *Rplp0* was used as reference gene. In **(B, D)** * and solid lines indicate significant, fixed 24-hour period cosine curve fit with cosinor analysis, whereas dashed lines show non-significant fit. Parameters of the fitted cosine curves are listed in **Supplementary Table 4**. ZT, *Zeitgeber* time.

Visceral adipose tissue is considered as a central hub integrating metabolic changes and inflammatory responses, best represented by increased production of proinflammatory cytokines in obesity and other metabolic disturbances (46, 54). Based on our above observations, we hypothesized that TRF alters both the rhythmic function and the adipokine production in the adipose tissue, which might influence systemic immune responses. Rhythmic operation of the visceral adipose tissue was investigated by following time-dependent changes in the expression of the core oscillator gene *per2*, one of the key components of the secondary feedback loop *reverbα*, and the fat-derived hormone *leptin* (55). Under TRF condition amplitude of the time-dependent changes of both *per2* and *leptin* were increased, whilst rhythmic expression of *reverbα* was phase shifted (**Figures 3A-D**; **Supplementary Table 4**), showing that TRF affected the entrainment of visceral adipose tissue. In addition, average *leptin* expression was ca. 30% lower in the TRF than in the AL group, suggesting that TRF has profound effects on the homeostasis of adipose tissue. Similarly to the gene expression levels – TRF reduced the average concentration and induced rhythmic changes of serum leptin as well (**Figures 3C, D**; **Supplementary Table 4**). This finding further supports the entraining effect of TRF on the metabolic rhythm of the adipose tissue.

**Figure 4:**
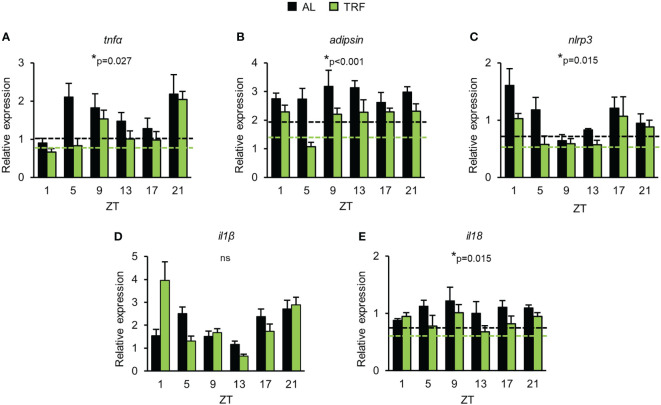
Time restricted feeding has anti-inflammatory effect in visceral adipose tissue. Relative mRNA expression of *tnfα* **(A)**, *adipsin* **(B)**, *nlrp3* **(C)**, *il1β* **(D)** and *il18* **(E)** in the course of day in visceral adipose tissue (mean + SEM, n = 3-9 (AL), n = 6-11 (TRF), two-way ANOVA, * indicates significant group effect). *Rplp0* was used as reference gene. Dashed lines indicate the average daily mRNA expression of the indicated gene, where significant group effect persists. ZT, *Zeitgeber* time ns, not significant.

As TRF alters the clock function of the adipose tissue and the circadian clock can directly control the activity of NFκB (9), we followed the daily expression pattern of the NFκB-dependent proinflammatory cytokine *tnfα*. Although *tnfα* expression did not display circadian rhythm, significant reduction in *tnfα* levels was observed in the TRF group compared to AL fed animals (**Figure 4A**). Adipsin (complement factor D) promotes lipid accumulation and differentiation of adipocytes and also activates the alternative pathway of the complement cascade, leading to the elevation of anaphylatoxin C3a production (56). As shown in **Figure 4B**, *adipsin* expression was lower in the TRF group than in the AL group. Literature data shows that the amount of the inflammasome regulator NLRP3 reflects the activity of NFκB and is sensitive to metabolic changes, like the concentration of LDL, cholesterol, fatty acids or ROS (56). Following stimulation, NLRP3 recruits caspase-1 to the inflammasome complex, which in turn cleaves and thereby activates the inflammatory cytokines IL1β and IL18. Although the expression of *il1β* did not differ between the groups, both *nlrp3 and il18* levels were decreased in TRF mice compared to AL fed animals (**Figures 4C-E**). In summary, our data indicate that despite the relatively short period of conditioning, TRF has a significant impact on the rhythm of metabolism and reduces inflammatory potential of the visceral adipose tissue.

### TRF affects time-dependent variations of circulatory leukocytes’ count

**Figure 5:**
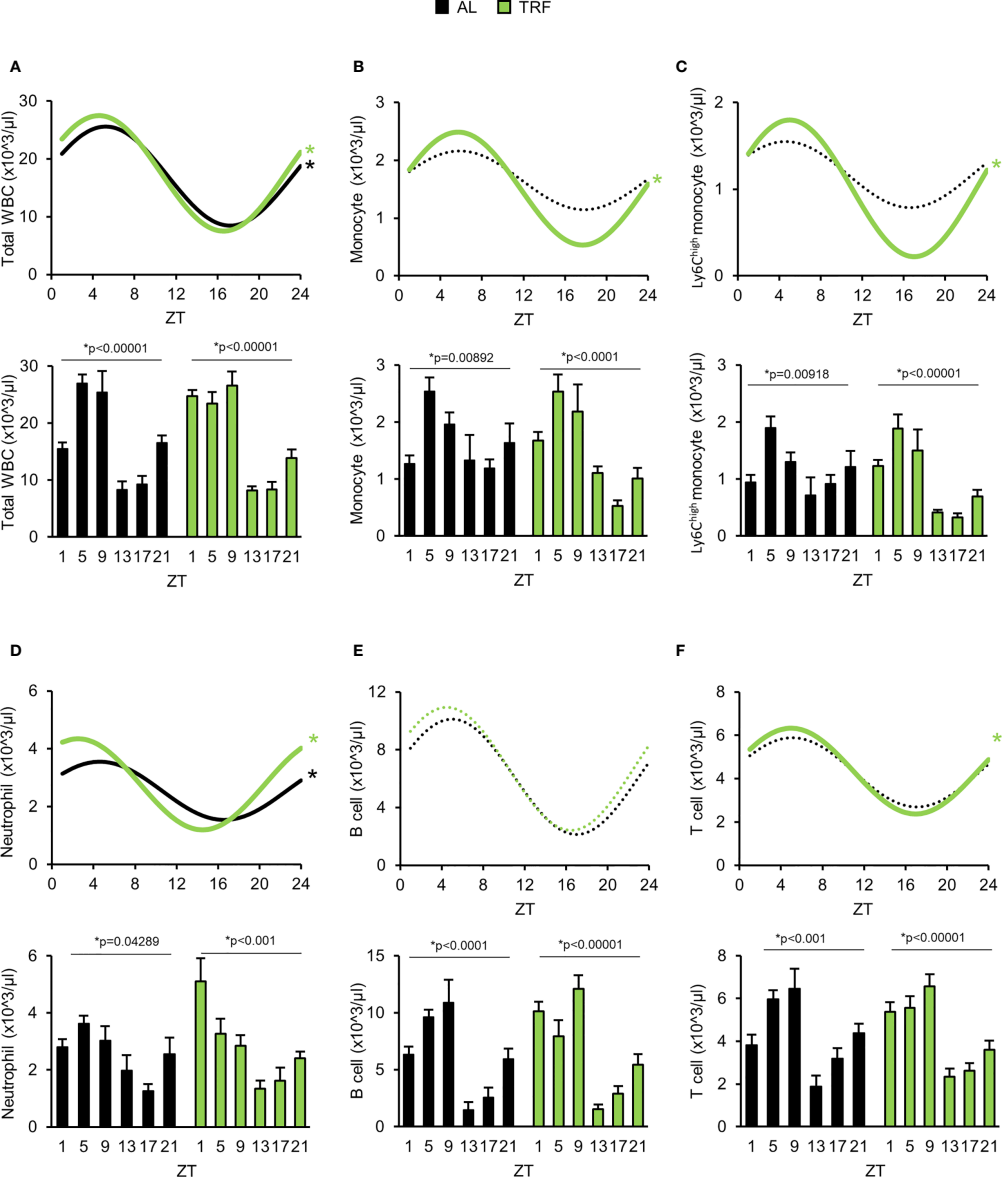
Time restricted feeding supports oscillation of the peripheral leukocyte counts. **(A-F)** Lower panels show peripheral leukocyte counts in the course of the day. Upper panels indicate cosine curve fit to data showed in lower panels. Total WBC **(A)**, monocyte **(B)**, Ly6C^high^ monocyte **(C)**, neutrophil **(D)**, B-lymphocyte **(E)** and T-lymphocyte **(F)** counts. Data information: In lower panels **(A–F)** data are presented as mean + SEM (n = 3-9 (AL) and n = 6-11 (TRF), one-way ANOVA, * indicates significant time effect). In upper panels * and solid lines indicate significant, fixed 24-hour period cosine curve fit with cosinor analysis, whereas dashed lines show non-significant fit. Parameters of the fitted cosine curves are listed in **Supplementary Table 5**. ZT, *Zeitgeber* time.

Leukocyte count in peripheral blood is controlled by the circadian clock. Number of the circulating cells depends on both leukocyte margination to the vessel wall and migration into the tissues and releasing from and homing back of the cells to the bone marrow, *i.e.* processes that are influenced by the circadian clock (27, 29, 57, 58). In line with this, we examined time-dependent changes of leukocyte counts in the peripheral blood and found larger difference between the minimal and maximal values in the TRF animals compared to the AL ones (**Figure 5A**; **Supplementary Table 5**; **Supplementary Figure 6**). Additionally, analysis of leukocyte subpopulations indicated that TRF had entraining effect on the rhythm of the cell numbers, with the most intense amplitude increase in monocyte and neutrophil counts (56% and 93% elevation, respectively) (**Figures 5B-F**; **Supplementary Table 5**). Further examination of the monocyte population revealed that the increased alteration of the Ly6C^high^ inflammatory monocyte counts was the main reason for the pronounced rhythm in the total monocyte counts (**Figure 5C**; **Supplementary Table 5**). In summary, our results raise the possibility that TRF has an impact on leukocyte trafficking between circulation and different tissues.

### TRF modulates the expression of adhesion molecules and cytokine receptors of peripheral neutrophils and monocytes and decreases the tissue leukocyte pool of the lung

Bone marrow functions – including coordination of homing and release of hematopoietic stem cells and neutrophils – is rhythmic (57, 59), thus an altered bone marrow activity could account for the pronounced leukocyte rhythm seen in the TRF group. Operation of the circadian clock in the bone marrow was investigated by following the expression *per2* and *reverbα* during a 24-hour period. Similarly to our observation in the visceral adipose tissue, in TRF mice the amplitude of *per2* RNA changes was increased, whereas rhythmic expression of *reverbα* was phase delayed (**Supplementary Figures 7A, B**; **Supplementary Table 6**), showing that TRF enhances circadian clock function of the bone marrow.

CXCL12 produced in the bone marrow is a main regulator of the time-of-day-dependent homing and release of neutrophils. Both its secretion and the expression of its receptor, CXCR4 are rhythmic (57). As recently reported, leptin can influence the production of CXCL12 *via* activation of LepR+ stromal cells of the bone marrow (60). As leptin showed rhythmic changes in the TRF animals, we investigated the expression of CXCL12 in the course of the day. Although clear daily variations were observed in the expression of CXCL12, no significant difference was found at either the RNA or the protein levels between the TRF and AL groups (**Supplementary Figures 7C, D**; **Supplementary Table 7**). In addition, at ZT1 and ZT13 serum levels of CXCL12 were also similar (**Supplementary Figure 7E**).

Low–grade inflammation or the activation of the NLRP3 inflammasome pathway in the bone marrow are also known to regulate the trafficking of cells with hematopoietic origin (61). Therefore, time-dependent expression of *tnfα*, *nlrp3*, *il1β*, *il18* RNA was investigated. In case of all RNA levels, significant time effect (two-way ANOVA, p<0.05) and daily variations were observed (**Supplementary Figure 7F**; **Supplementary Table 7**), however, with no differences between the TRF and AL groups (**Supplementary Figure 7F**). In summary, our data suggest that the altered rhythm of blood neutrophil count is unlikely originated from differences in the expression of bone marrow derived factors.

**Figure 6:**
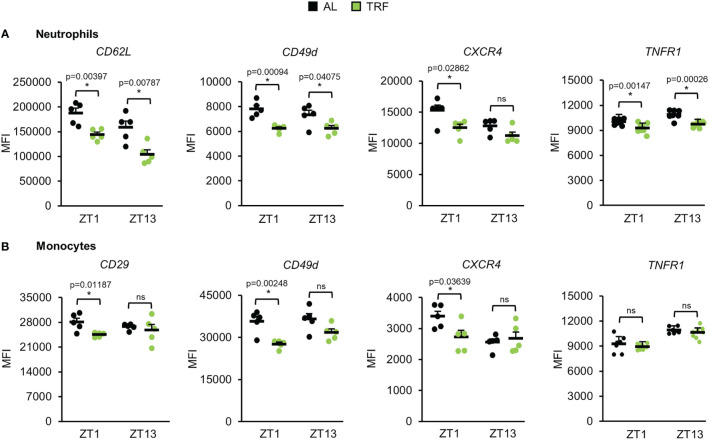
Time restricted feeding modulates the expression of adhesion molecules and cytokine receptors of peripheral neutrophils and monocytes. Selectin (CD62L), integrin (CD29, CD49d) and cytokine receptor (CXCR4, TNFR1) expression of neutrophils **(A)** and monocytes **(B)** at ZT1 and ZT13 (mean + SEM, n = 5 (AL), n = 5 (TRF), two-sample t-test, * p < 0.05). ZT, *Zeitgeber* time. Histograms of fluorescence intensities are shown in **Supplementary Figure 9** ns, not significant.

Migration of white blood cells to tissues shows circadian rhythm which is dependent on the daily oscillation of leukocyte activating cytokines and time-dependent changes in endothelial functions (58). Modified expression of adhesion molecules and cytokine receptors on both neutrophils and monocytes and altered migration ability of the cells could account for rhythm changes in the leukocyte counts and could also mark the inflammatory potential of the cells. Therefore, the cell surface levels of molecules involved in selectin signaling (CD62L, CD162), integrins (CD11a, CD11b, CD29, CD49d), and cytokine receptors (CXCR2, CXCR4, TNFR1) of neutrophils and monocytes were compared between the TRF and the AL groups at the beginning of the inactive phase (ZT1) and at the beginning of the active phase (ZT13) (**Figure 6**; **Supplementary Figures 8**, **9**). In neutrophils of the TRF group significantly lower surface levels of CD62L, CD49d and TNFR1 were detected at both time points compared to the cells of the AL group. In addition, CXCR4 showed reduced expression in TRF neutrophils, but the difference reached the significance level only at ZT1 (**Figure 6A**; **Supplementary Figures 8A**, **9A**). In monocytes, significant reduction in the cell surface expression of CD29, CD49d and CXCR4 was measured at ZT1 (**Figure 6B**; **Supplementary Figures 8B**, **9B**).

According to literature data, leptin activates various immune functions, including the adhesion and migration ability of leukocytes [for review see (62)]. To test whether elevated leptin levels in the AL group could contribute to the increased expression of cell surface structures in neutrophils and monocytes, we treated blood samples with leptin and analyzed the expression of adhesion molecules that were affected by TRF in the previous experiment. Leptin applied in a concentration comparable to maximal serum levels in the AL samples induced the expression of CD49d on both neutrophils and monocytes and the expression of CXCR4 on monocytes (**Supplementary Figure 10**). These results suggest, that leptin could mediate, at least partially, the effect of adipose tissue on leukocyte responsiveness in a feeding-dependent manner.

**Figure 7:**
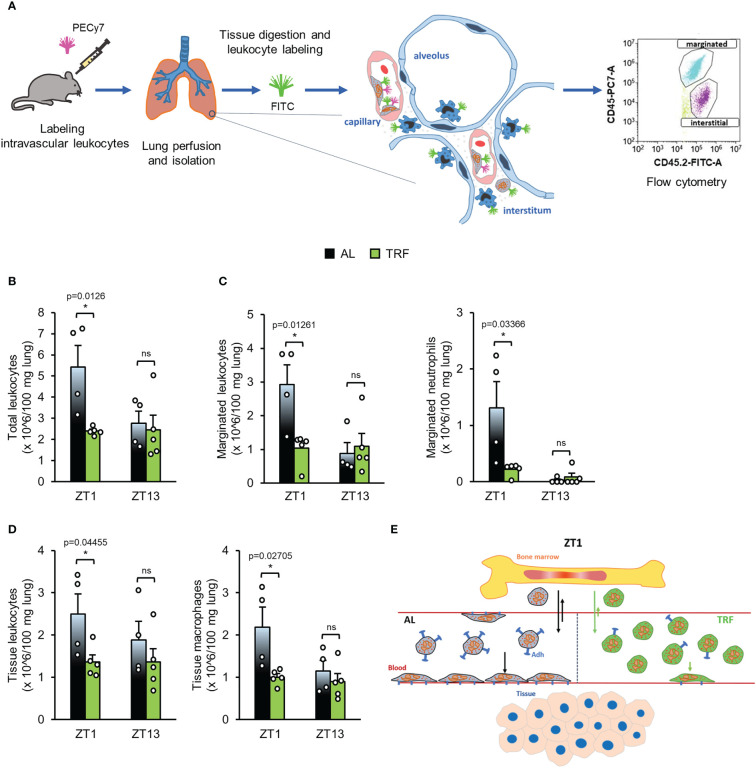
Time restricted feeding decreases the migration of leukocytes to the lung. **(A)** Experimental setup to the identification of leukocyte populations in the lungs. For further details see Materials and Methods. **(B-D)** Leukocyte populations in the lungs: total leukocyte **(B)**, marginated (adhered) leukocyte and neutrophil **(C)**, tissue leukocyte and macrophage **(D)** counts in the lungs at ZT1 and ZT13 (mean + SEM, n = 4 (AL) and n = 5 (TRF), two-sample t-test, * p < 0.05). **(E)** Time-of-the-day-dependent changes of neutrophil counts in blood. Abundance of circulating neutrophils is determined by rhythmic alterations of cell trafficking between blood and bone marrow and intensity of margination to the endothelial surface and migration from the intravascular space into the tissues. Arrows indicate the direction and intensity of leukocyte movements in the AL (black) and TRF (green) groups, respectively. The model suggests that the lower expression of adhesion molecules in the TRF group weakens the margination capacity resulting in significantly increased neutrophil count in blood during the inactive phase of mice. Adh, Adhesion molecule ns, not significant.

The obtained differences between the AL and TRF groups in the expression of adhesion molecules and cytokine receptors raise the possibility that TRF impacts both the margination (adherence to the vessel wall) and tissue accumulation of the cells. We used a method that allows distinguishing between marginated and tissue leukocytes in the lung, and compared the leukocyte pools between the AL and the TRF groups at ZT1 and ZT13 (**Figure 7A**). As shown in **Figure 7B**, the total leukocyte count in the lung halved at ZT1 in the TRF fed animals, suggesting a remarkable effect of TRF on leukocyte recruitment. In the TRF animals the marginated leukocyte pool was significantly reduced at ZT1, along with a markedly decreased neutrophil count compared to the AL fed mice (**Figure 7C**). Also, in case of tissue leukocytes lower counts were detected in the TRF group at ZT1, which could be linked to the reduced macrophage population (**Figure 7D**).

Based on the differences in the spleen weight we compared the abundance of spleen neutrophils in the samples of AL and TRF animals **(** **Supplementary Figure 11** **)**. The higher neutrophil ratio obtained in the AL samples at ZT13 might also reflect an enhanced migration ability of the AL cells compared to the TRF ones.

In summary, the lower expression of adhesion molecules and chemokine receptors in leukocytes of the TRF group correlates with a decreased tissue leukocyte pool and increased number of circulating neutrophils, which could imply a lower inflammatory potential of tissues.

## Discussion

Temporal restriction of food intake to the active phase of behaviour was reported to enhance metabolic fitness in both animal and human studies, and was found to be beneficial in the prevention and treatment of obesity and associated metabolic diseases (63). In mice, detailed analysis of metabolic parameters, liver functions in particular, revealed that TRF strengthens the oscillation of the peripheral tissue clock and thereby the rhythmic expression of metabolic regulators which might lead to a better and thus more efficient timely control of metabolism (22). Although it is well documented that metabolism and immune functions interact, the mechanistic connections between the two systems are only partially understood. It is especially unexplored whether metabolic rhythm and its modifications can influence immune cells and complex immune responses. In this study we show that even a 4-week period with temporal regulation of feeding significantly affects immune functions at both the systemic and cellular level. Compared to animals with *ad libitum* food availability, in mice subjected to TRF in the active phase, a less severe form of autoimmune arthritis was developed and substantial differences in both the circulating and the tissue pool of leukocytes were detected. Time-of-the-day-dependent changes in neutrophil abundance in the blood are dependent on rhythmic alterations of cell trafficking to and from the bone marrow. During the inactive (light) phase homing of aged neutrophils back to the bone marrow becomes dominant and leads to a decrease of peripheral cell count, whereas later in the active phase egress of young cells from the bone marrow results in elevation of the peripheral neutrophil number (57). TRF increased the amplitude of time-dependent changes of clock genes in the bone marrow, and the enhanced rhythmic function of bone marrow cells might be reflected by a higher amplitude of leukocyte homing and egress. Although expression of bone marrow derived factors was similar in TRF and AL mice, the reduced expression of CXCR4 in TRF neutrophils compared to AL cells could result in higher neutrophil count in the circulation at ZT1. The decreased expression of adhesion molecules might lower the level of marginated and tissue resident leukocytes as well and thus contribute to the elevation of neutrophil count in the blood and the lower accumulation of the cells in the arthritic limb of the TRF animals at the beginning of the light (inactive) phase (**Figure 7E**). However, margination and migration of leukocytes is also dependent on the expression of endothelial structures interacting with leukocyte receptors. Importantly, endothelial ICAM-1 and VCAM-1 levels display daily oscillation which results in enhanced tissue accumulation of leukocytes during the active (dark) phase (58). Whether TRF affects expression of these endothelial structures, needs further investigations.

Neutrophil infiltration follows a tight circadian pattern (64), which contributes to a balanced immune homeostasis of the tissue but might influence pathological reactions as well. During infections, augmented neutrophilia and disturbed rhythmicity of the cell trafficking could lead to excessive pulmonary damage (65). Thus, our results raise the possibility that TRF could have protective effect and may prevent overactivation of the immune cells. In addition, the increased macrophage population detected in the lung tissue may also contribute to an elevation of the inflammatory potential in the AL group. Enhanced tissue accumulation of both monocytes and neutrophils is often associated with a more severe prognosis of diseases with excessive tissue damage, such as autoimmune arthritis, acute pancreatitis or the SARS-CoV2 infection of the lung (66). It is important to note, that the right timing of immune activation is crucial not only in the prevention of tissue damage and autoinflammatory processes, but also in the effective elimination of pathogens. In line with this, a recent study showed that night-restricted feeding can enhance cytokine production in response to bacterial endotoxin treatment (67).

Although basic metabolic indicators did not differ between TRF and AL mice, rhythm and function of visceral adipocytes were substantially affected by temporal variations of food intake. Amplitude of the time-dependent changes in the expression of both leptin and core clock components was increased by TRF and the average leptin level throughout the day was markedly reduced in both the adipose tissue and the serum. In addition, TRF attenuated inflammatory activity of adipose tissue, as reflected by the reduction of the expression of the proinflammatory cytokines *tnfα* and *il18*, *adipsin* and the inflammasome constituent *nlrp3*. We propose that visceral adipose tissue can act both as a sensitive indicator of metabolism and as a mediator between TRF-induced metabolic changes and immune system responsiveness. Adipsin was recently reported to play pivotal role in the development of K/BxN serum-induced arthritis (68). Complement activation, particularly the alternative complement pathway is central in the pathology of the effector phase of rheumatoid arthritis (51) and adipsin is an important contributor of this pathway by activating the C3 convertase. Complement activation may promote neutrophil infiltration into the joint where activated neutrophils further trigger the complement pathway, leading to an amplification of the inflammatory process (68). Leptin was also considered to play critical role in the pathology of human rheumatoid arthritis (69). As a potent modulator of multiple levels of immune responses, it might affect both innate and adaptive immune processes. It was described as an effective stimulator of almost all types of immune cells, including neutrophils and monocytes which both showed altered time-dependent abundance under TRF conditions. In addition, our results indicate that leptin increases the expression of adhesion molecules on both cell types. Leptin is also considered a survival signal of neutrophils, as it delays apoptosis of mature neutrophils (70). Furthermore, exogenous leptin induces neutrophil migration through TNFα and CXCL1 dependent mechanisms (71). Leptin is a sensitive indicator of the nutrition state and short-term treatment of starved animals with physiological amounts of leptin was found to increase spleen weight (72). The detected changes in the leptin expression and serum leptin levels in response to TRF could be responsible for the reduced spleen mass under our experimental conditions as well. Overall, higher leptin levels produced by the adipose tissue of AL fed mice as compared to the TRF group may contribute to the enhanced migration capacity and reactivity of neutrophils in our experiments. In addition, leptin can also be produced by bone marrow adipocytes and if reaching high local concentration, it stimulates myeloid differentiation which can promote leukocytosis often found in obesity (73). Other indicators of the inflammatory state - such as activation of NLRP3 and elevation of pro-inflammatory cytokine levels in the adipose tissue and higher expression of the cytokine receptor TNFR1 in neutrophils – might also contribute to the enhanced inflammatory potential observed in AL fed mice (74). In addition, NLRP3 was found to be a stimulating factor of tissue infiltration of myeloid cells (69, 75).

Finally, it is important to note, that in this study we used a moderate form of TRF with only 14 hours fasting phase and for a relatively short period (4 weeks). This feeding regimen corresponds to the feeding habit of the animals in nature and it did not cause serious stress. Therefore, this feeding schedule can serve as a good experimental model for a balanced and attainable eating pattern which might contribute to the prevention and therapy of human inflammatory processes and metabolic diseases.

## Data availability statement

The original contributions presented in the study are included in the article/**Supplementary Materials**. Further inquiries can be directed to the corresponding authors.

## Ethics statement

The animal study was reviewed and approved by Animal Experimentation Review Board of the Semmelweis University and the Government Office for Pest County (Hungary) (Ethical approval: PE/EA/1967-2/2017).

## Author contributions

KE and KK designed and supervised the project. KE and ÁS designed the experiments. KE, ÁS, ZB, BK, ÁK performed the experiments and analyzed the data. KE, ÁS and KK interpreted the results and wrote the manuscript. AM gave technical and financial support to the revised version of the manuscript. All authors contributed to the article and approved the submitted version.

## Funding

This work was supported by the National Research, Development and Innovation Office – NKFIH (K115953 and K132393), the Ministry of Innovation and Technology of Hungary (National Research, Development and Innovation Fund, financed under the projects TKP2021-EGA-25 and TKP2021-EGA-24), inhouse grants of the Semmelweis University (STIA-2017 to KK and STIA-2018 to KE) and by the ÚNKP-20-4-II-SE-23 New National Exellence Program of the Ministry for Innovation and Technology from the source of the National Research, Development and Innovation Fund to KE. KE is a Merit Scholar of the Semmelweis University.

## Acknowledgments

We thank Rita Krisztina Nagy and Zalán Lumniczky for the excellent technical assistance. We are especially grateful to Bence Erdős for technical support.

## Conflict of interest

The authors declare that the research was conducted in the absence of any commercial or financial relationships that could be construed as a potential conflict of interest.

## Publisher’s note

All claims expressed in this article are solely those of the authors and do not necessarily represent those of their affiliated organizations, or those of the publisher, the editors and the reviewers. Any product that may be evaluated in this article, or claim that may be made by its manufacturer, is not guaranteed or endorsed by the publisher.
