# Supplementary material for: Time restricted feeding modifies leukocyte responsiveness and improves inflammation outcome
Source: Front Immunol. 2022 Nov 2;13:924541. doi: 10.3389/fimmu.2022.924541 (PMC9666763; doi:10.3389/fimmu.2022.924541)
Supplement: Supplementary file 1 [file DataSheet_1.pdf]

Supplementary Material

Supplementary Figure 1. Flow cytometric analysis of leukocyte populations in blood.

Supplementary Figure 2. Flow cytometric analysis of migratory factors expressed on neutrophils and monocytes.

Supplementary Figure 3. Flow cytometric analysis of the marginated and tissue leukocyte populations of the lung.

Supplementary Figure 4. Adrenal mass, serum corticosterone levels, body weight, food intake and glucose tolerance of the experimental groups.

Supplementary Figure 5. Body weight of the animals did not differ in arthritis experiments.

Supplementary Figure 6. Peripheral leukocyte counts did not differ before conditioning the animals to different feeding regimens.

Supplementary Figure 7. Time restricted feeding entrains the peripheral clock but does not modify the rhythm of CXCL12 and the inflammatory state of the bone marrow.

Supplementary Figure 8. Expression of migratory factors of neutrophils and monocytes.

Supplementary Figure 9. Histograms of fluorescence intensities presented in Figure 6.

Supplementary Figure 10. Expression of migratory factors of neutrophils and monocytes after leptin treatment.

Supplementary Figure 11. Effect of TRF on the abundance of neutrophils among spleen leukocytes.

Supplementary Table 1. Primer and probe sequences for gene expression analysis.

Supplementary Table 2. Antibodies and staining reagents.

Supplementary Table 3. Parameters of the fitted cosine curves shown in Figure 2B.

Supplementary Table 4. Parameters of the fitted cosine curves shown in Figure 3B and D.

Supplementary Table 5. Parameters of the fitted cosine curves shown in Figure 5.

Supplementary Table 6. Parameters of the fitted cosine curves shown in Supplementary Figure 7B.

Supplementary Table 7. Parameters of the cosine curves fitted to the data shown in Supplementary Figure 7C, D, F.

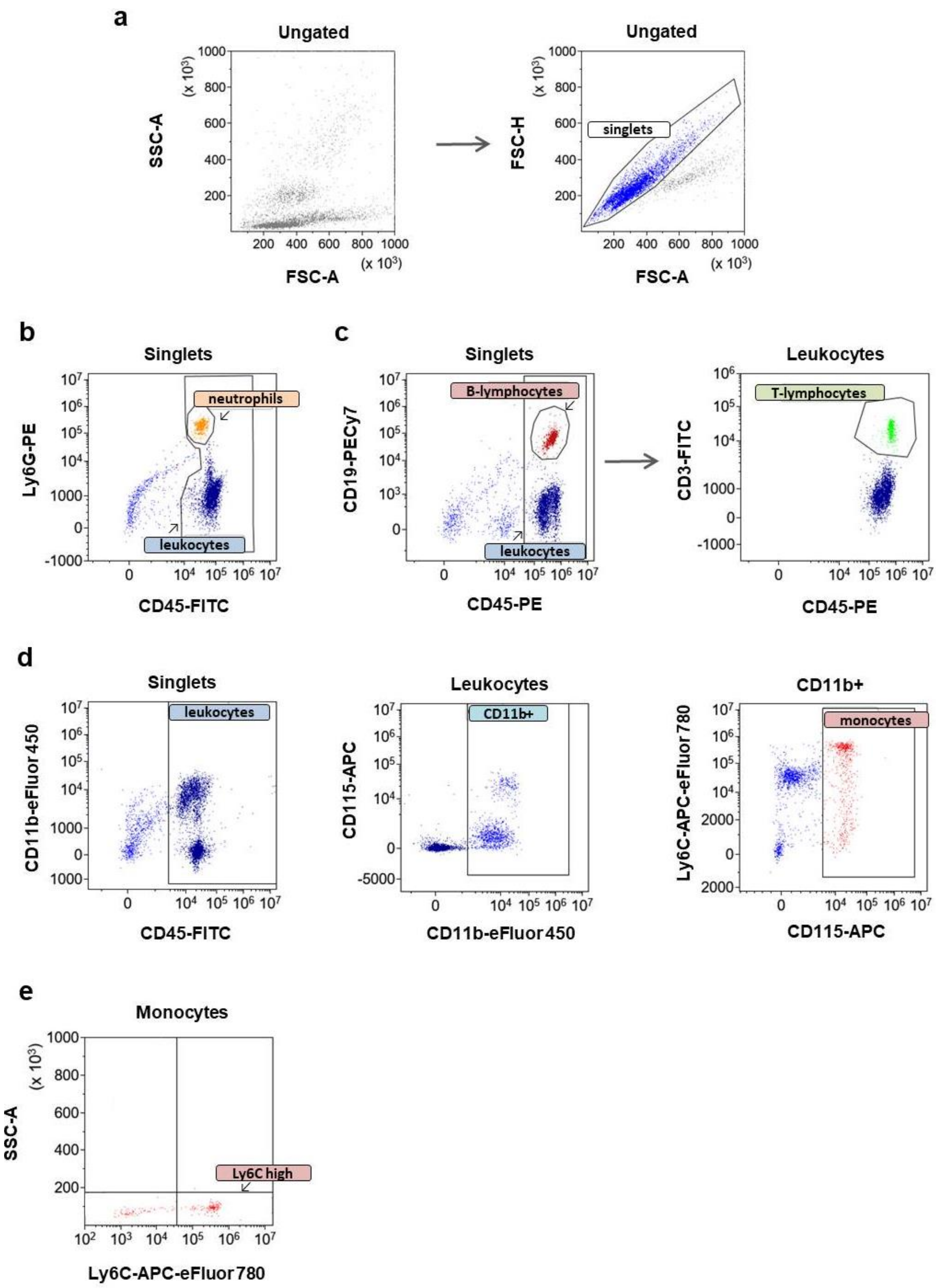

Supplementary Figure 1. Flow cytometric analysis of leukocyte populations in blood. Before analysis of specific labelings, singlets were gated in all cases (**A**). Identification of neutrophils (**B**), B- and T-lymphocytes (**C**), monocytes (**D**) and Ly6C^{high} inflammatory monocytes (**E**).

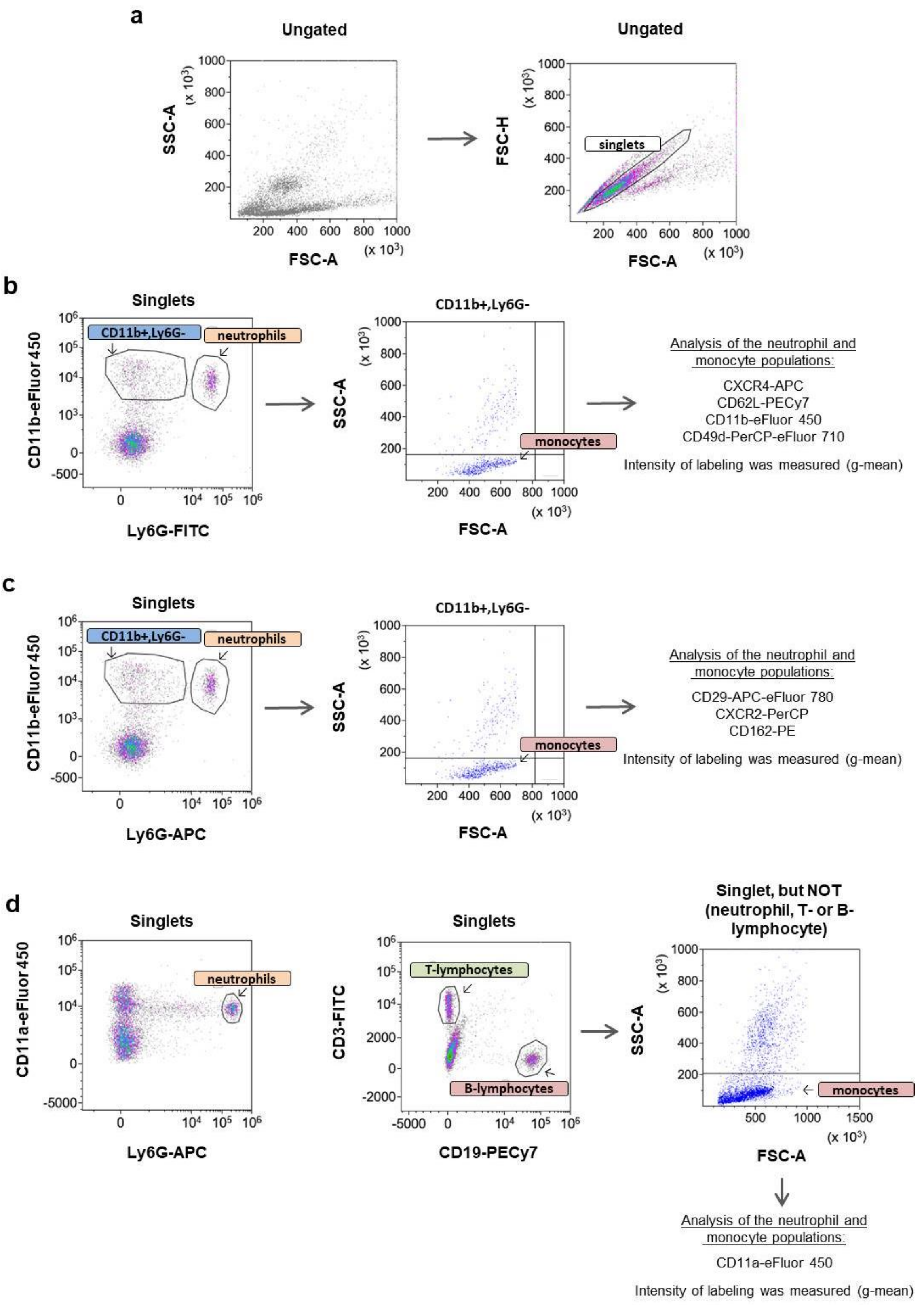

Supplementary Figure 2. Flow cytometric analysis of migratory factors expressed on neutrophils and monocytes. Before analysis of specific labelings, singlets were gated in all cases (**A**). Identification of neutrophils (Ly6G+) and monocytes (CD11b+, Ly6G-, SSC^{low} or Ly6G-, CD3-, CD19-, SSC^{low}) (**B, C, D**). Intensity of labeling signals was determined using g-mean values.

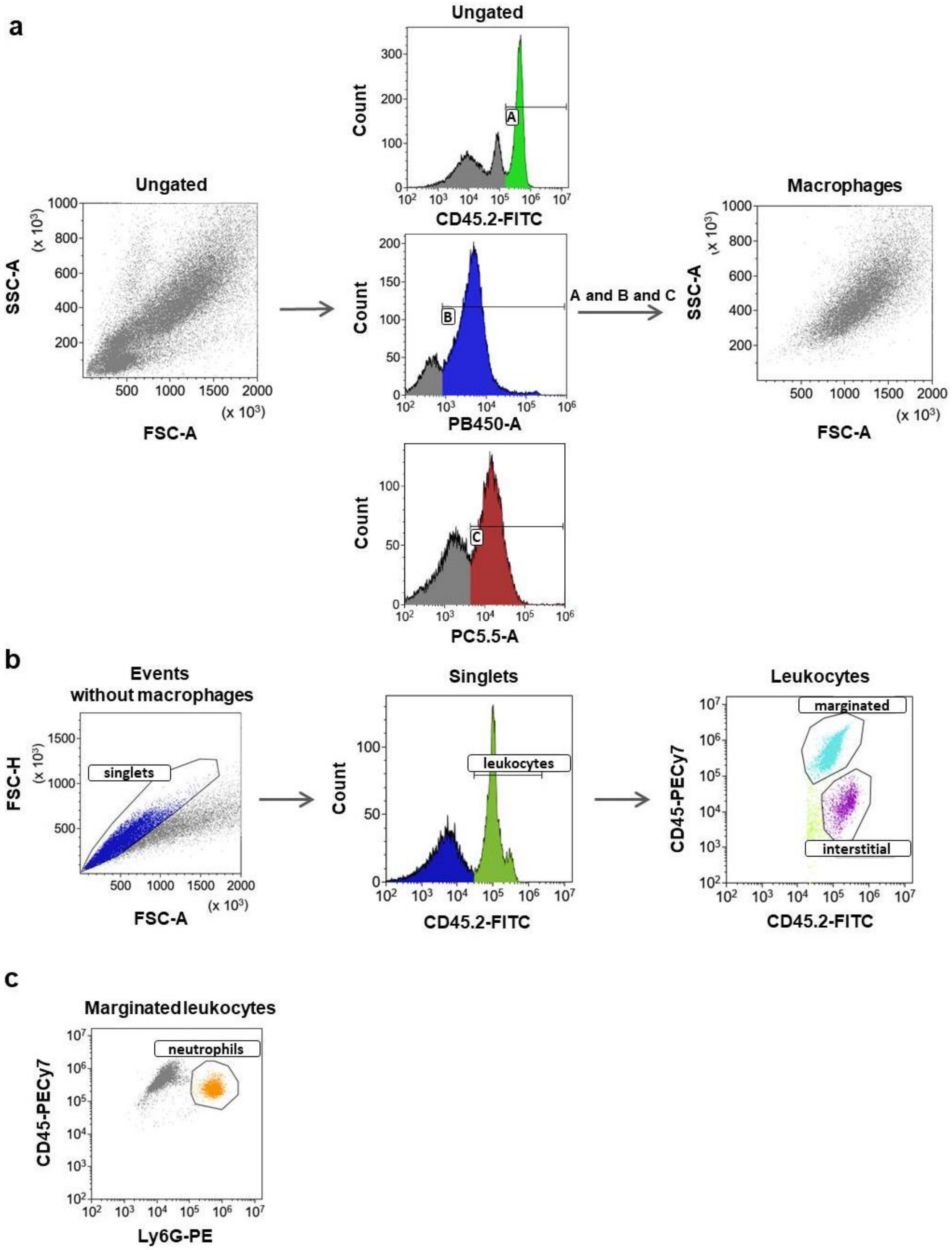

Supplementary Figure 3. Flow cytometric analysis of the marginated and tissue leukocyte populations of the lung. (**A**) Identification of tissue macrophages according to CD45-FITC positivity and their high autofluorescence measured in PB450 and PC5.5 channels. (**B**) Identification of the marginated (CD45-PECy7+, CD45.2-FITC+) and tissue leukocytes (CD45-PECy7-, CD45.2-FITC+) of the lung. (**C**) Identification of marginated neutrophils (Ly6G-PE+) of the lung.

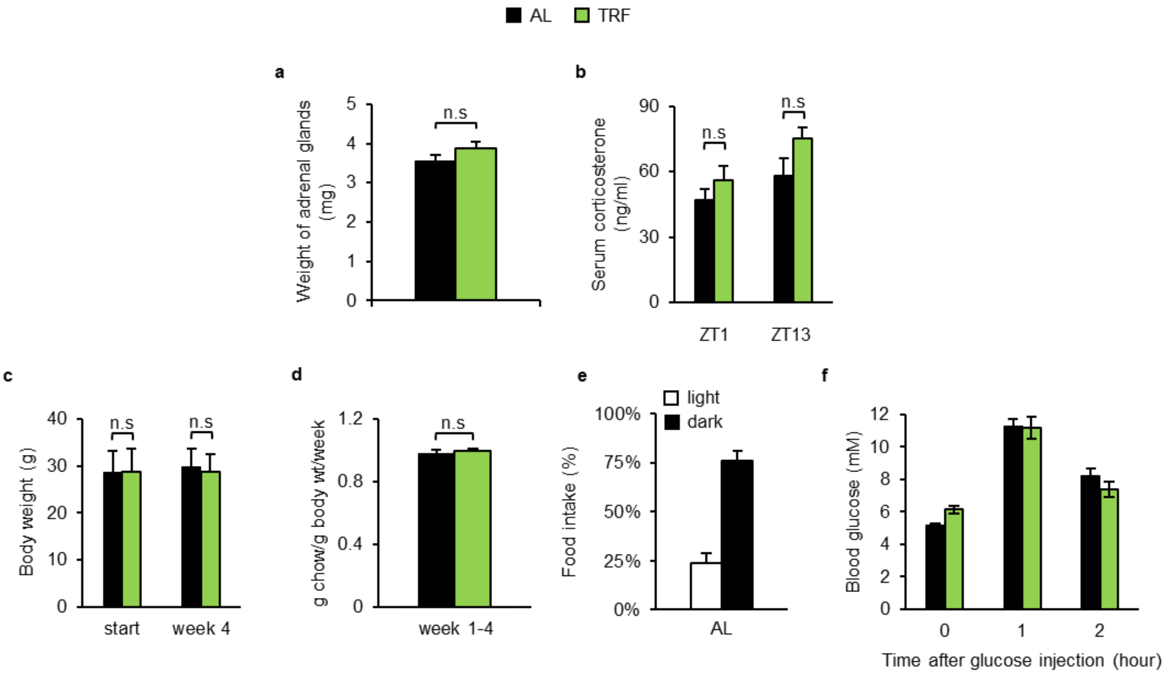

Supplementary Figure 4. Adrenal mass, serum corticosterone levels, body weight, food intake and glucose tolerance of the experimental groups. (**A**) Adrenal mass after 4-week time restricted feeding (mean + SD n=15 (AL), n=13 (TRF), two-sample t-test, n.s. p=0.154). (**B**) Serum corticosterone levels after 4-week time restricted feeding (mean + SEM n=11 (AL), n=11 (TRF), two-sample t-test, n.s. p=0.287 and p=0.091 for ZT1 and ZT13 respectively). (**C**) Body weight of the animals at the start and after 4 weeks of the feeding regimens (mean + SD, n=75 (AL), n=82 (TRF), two-sample t-test, n.s. p=0.871 and p=0.137 for start and week 4 respectively). (**D**) Average of the weekly food intake normalized to g body weight (mean + SEM, n=24 (AL), n=28 (TRF), two-sample t-test, n.s. p=0.571). (**E**) Food intake of the ad libitum fed animals in the light and dark phases of the day (mean + SD n=5 cages) (**F**) IPGTT measured in AL and TRF mice after 16 hours fasting. Average blood glucose levels after IPGTT in AL and TRF mice (mean ± SEM, n=7 (AL), n=9 (TRF), repeated measures ANOVA, time effect p<0.001, group effect p=0.986). ZT=*Zeitgeber* time, n.s=not significant

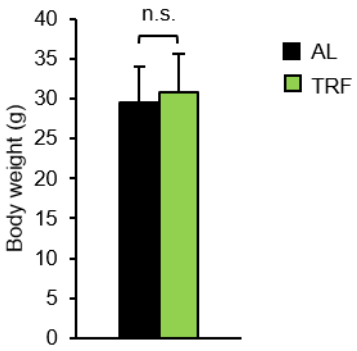

Supplementary Figure 5. Body weight of the animals did not differ in arthritis experiments. Body weight of the animals at the induction of K/BxN serum transfer arthritis (mean + SD, n=25 (AL), n=23 (TRF), two-sample t-test, n.s. p=0.348). n.s=not significant

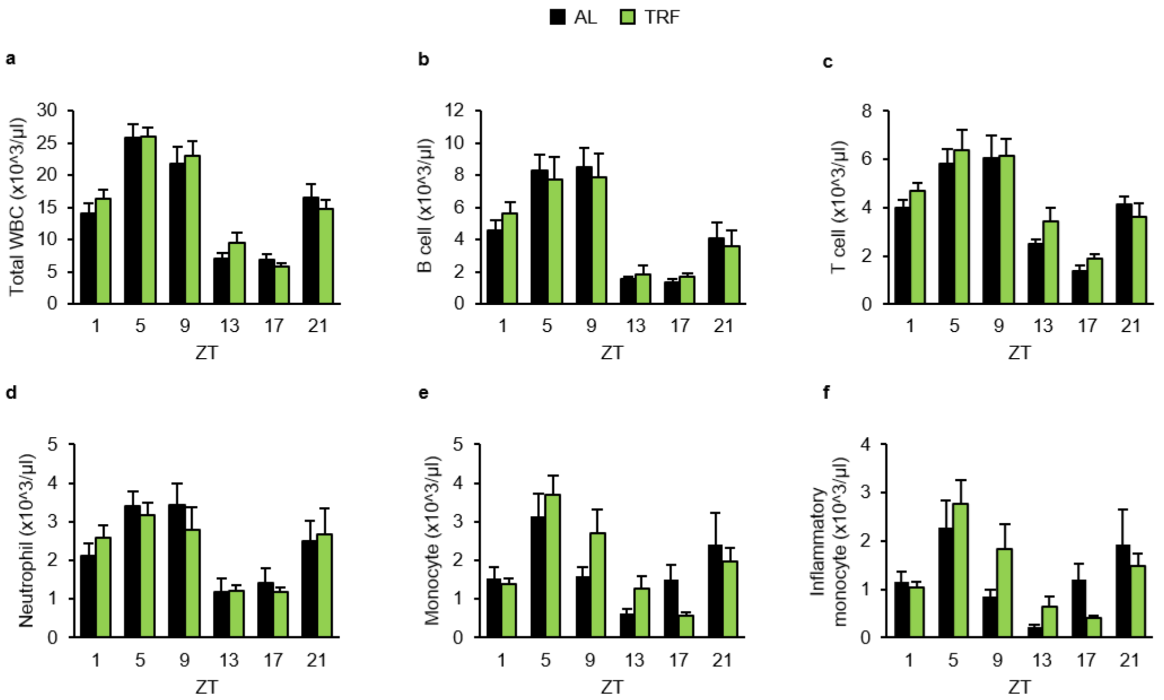

Supplementary Figure 6. Peripheral leukocyte counts did not differ before conditioning the animals to different feeding regimens. Total WBC (**A**), B-lymphocyte (**B**), T-lymphocyte (**C**), neutrophil (**D**), monocyte (**E**) and Ly6C^{high} inflammatory monocyte (**F**) counts on the starting day of the 4-week feeding regimens (mean ± SEM, n=3-9 (AL), n=6-11 (TRF), two-way ANOVA, n.s.). ZT=*Zeitgeber* time, n.s=not significant

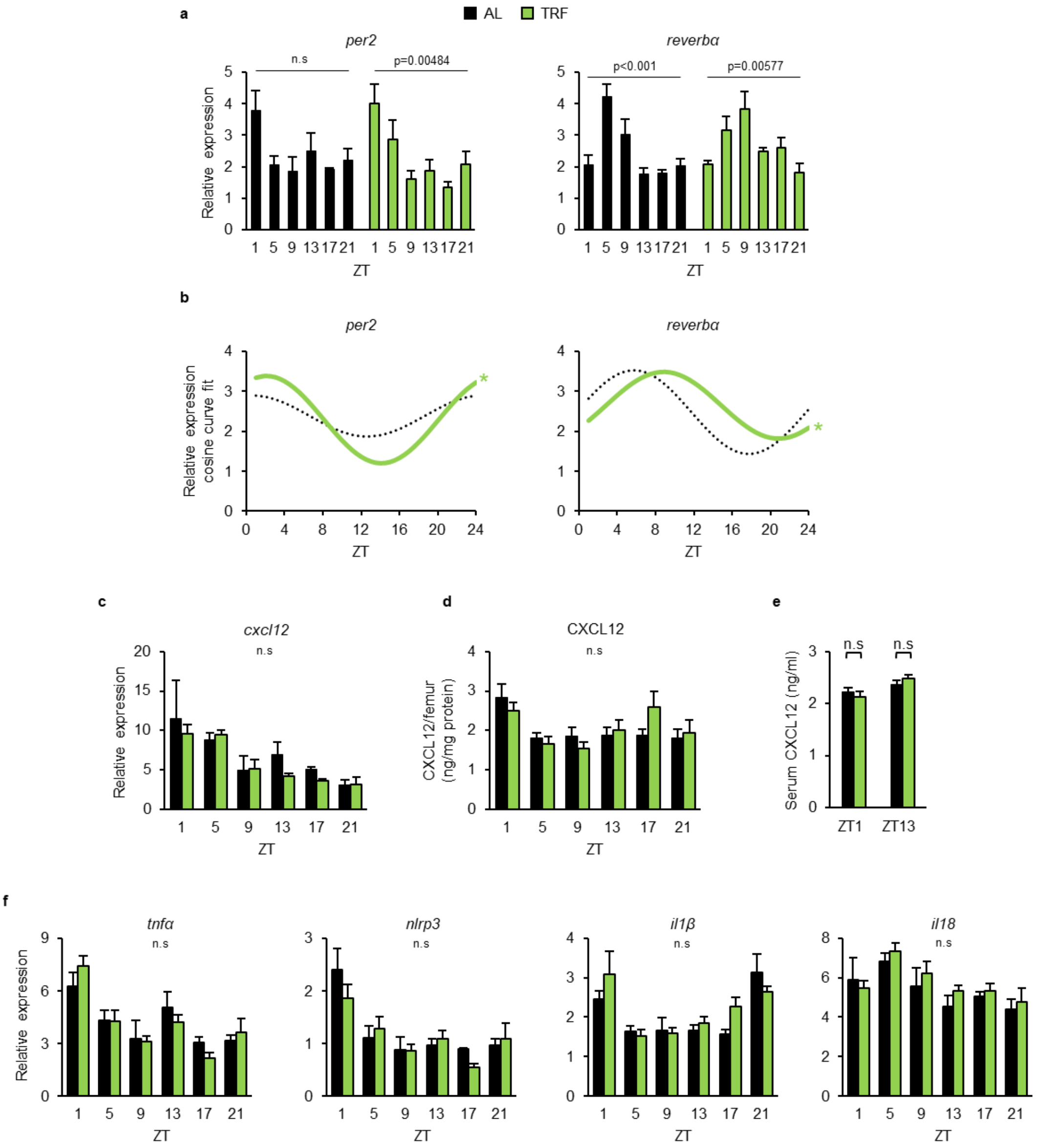

Supplementary Figure 7. Time restricted feeding entrains the peripheral clock but does not modify the rhythm of CXCL12 and the inflammatory state of the bone marrow. (**A**) Relative mRNA expression of *per2* and *reverbα* in the course of the day (mean + SEM, n=3-8 (AL), n=6-9 (TRF), one-way ANOVA, * indicates significant time effect). *Rplp0* was used as a reference. (**B**) Cosine curve fit to relative expression data showed in (**A**), * and solid lines indicate significant, fixed 24-hour period cosine curve fit with cosinor analysis, whereas dashed lines show non-significant fit. Parameters of the fitted cosine curves are listed in Supplementary Table 6. (**C**) Relative mRNA expression of *cxcl12* (mean + SEM, n=3-8 (AL), n=6-9 (TRF), two-way ANOVA, time effect p=0.005, group effect n.s.). *Rplp0* was used as a reference. (**D**) Protein expression of CXCL12 in the bone marrow (mean ± SEM, n=5-10 (AL), n=7-11 (TRF), two-way ANOVA, time effect p=0.007, group effect n.s.). (**E**) Serum concentrations of CXCL12 (mean + SEM, n=4-8 (AL), n=3-11 (TRF), two-sample t-test, n.s.). (**F**) Relative mRNA expression of *tnfα*, *nlrp3*, *il1β* and *il18* in the course of day in bone marrow (mean + SEM, n=3-8 (AL) and n=6-9 (TRF), two-way ANOVA, group effect n.s.). *Rplp0* was used as a reference. ZT=*Zeitgeber* time, n.s=not significant

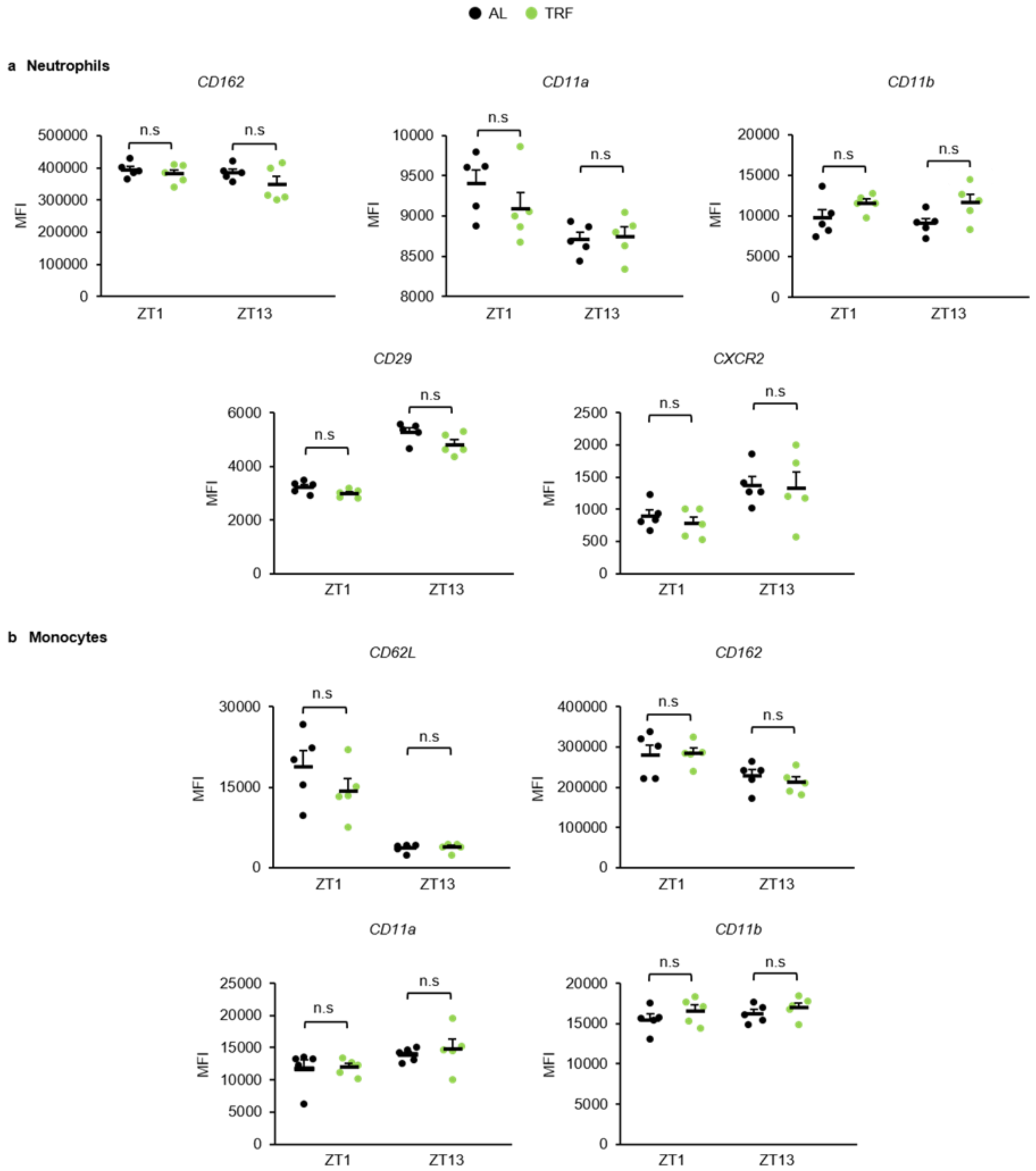

Supplementary Figure 8. Expression of migratory factors of neutrophils and monocytes. Selectin (CD62L), selectin ligand (CD162), integrin (CD11a, CD11b, CD29) and chemokine receptor (CXCR2) expression of neutrophils (**A**) and monocytes (**B**) at ZT1 and ZT13 (mean + SEM, n=5 (AL), n=5 (TRF), two-sample t-test, *p<0.05). ZT=*Zeitgeber* time, n.s=not significant

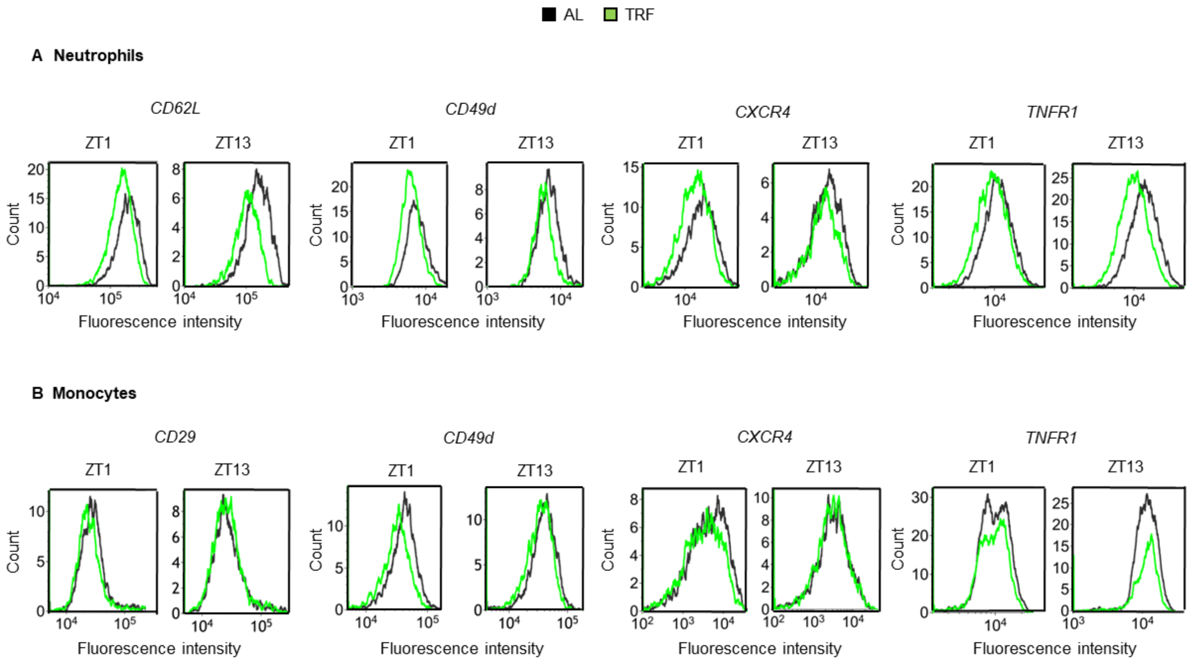

Supplementary Figure 9. Histograms of fluorescence intensities presented in Figure 6. Selectin (CD62L), integrin (CD29, CD49d) and cytokine receptor (CXCR4, TNFR1) expression of neutrophils (**A**) and monocytes (**B**) at ZT1 and ZT13. ZT=*Zeitgeber* time

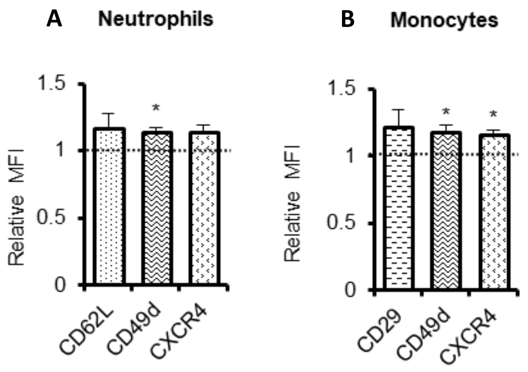

Supplementary Figure 10. Expression of migratory factors of neutrophils and monocytes after leptin treatment. Selectin (CD62L), integrin (CD29, CD49d) and chemokine receptor (CXCR4) expression of neutrophils (**A**) and monocytes (**B**) after leptin treatment compared to vehicle (mean + SEM, n=5, one-sample t-test, *$p<0.05$).

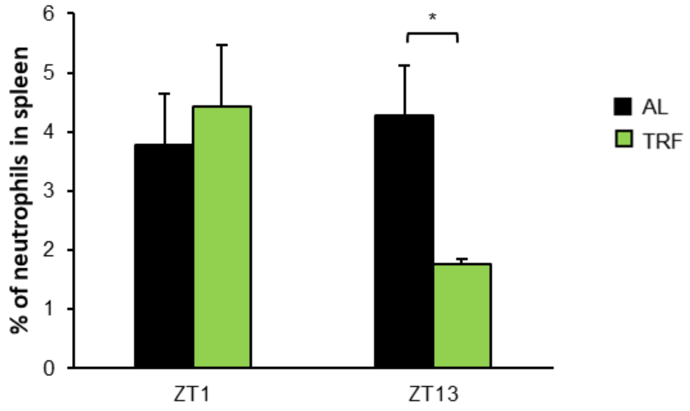

Supplementary Figure 11. Effect of TRF on the abundance of neutrophils among spleen leukocytes. Percentage of neutrophils among spleen leukocytes at ZT1 and ZT13 (mean + SEM, n=3-6 (AL) and n=5-8 (TRF), two-sample t-test, *p<0.05). ZT=*Zeitgeber* time

Supplementary Table 1.

Primer and probe sequences for gene expression analysis.

gene		sequence
rplp0	forward	5'-CTCGCTTTCTGGAGGGTGTC-3'
	reverse	5'-AGTCTCCACAGACAATGCCA-3'
	probe	5'FAM-TGCCTCGGTGCCACACTCCA-TAMRA3'
per2	forward	5'-GCTTCCGCCCTGGAGTGT-3'
	reverse	5'-TCTCCTCCATGCACTCCTGAG-3'
	probe	5'FAM-CAGCGGCTTAGATTC-MGB3'
reverbα	forward	5'-GACCTTTCTCAGCACGACC-3'
	reverse	5'-CATCACTGTCTGGTCCTTCAC-3'
	probe	5'FAM-CAAAGCGCACCATCAGCACCTC-TAMRA3'
cxcl12	forward	5'-CGCTCTGCATCAGTGACG-3'
	reverse	5'-TGAAGGGCACAGTTTGGAG -3'
	probe	5'FAM-CTTGACGTTGGCTCTGGCGATGT-TAMRA3'
tnfα	forward	5'-CCCTCCAGAAAAGACACCATG-3'
	reverse	5'-GCCACAAGCAGGAATGAGAAG -3'
	probe	5'FAM-CACAGAAAGCATGATCCGCGACG-TAMRA3'
il1β	forward	5'-TCCTGTGTAATGAAAGACGGC-3'
	reverse	5'-ACTCCACTTTGCTCTTGACTTC-3'
	probe	5'FAM-TTGGGTATTGCTTGGGATCCACACTC-TAMRA3'
il18	forward	5'-GCCTCAAACCTTCCAAATCAC-3'
	reverse	5'-GTTGTCTGATTCCAGGTCTCC-3'
	probe	5'FAM-TGCCATGTCAGAAGACTCTTGCGT-TAMRA3'
nlrp3	forward	5'-ATGGGTTTGCTGGGATATCTC-3'
	reverse	5'-GCGTTCCTGTCCTTGATAGAG-3'
	probe	5'FAM-AGAACCTGCTTCTCACATGTCGTCTG-TAMRA3'
leptin	forward	5'-AGCCTCACTCTACTCCACAG-3'
	reverse	5'-CCTCTACATGATTCTTGGGAGC-3'
	probe	5'FAM-TCAGCATTCAGGGCTAACATCCAACT-TAMRA3'
adipsin	forward	5'-CCTGAACCCTACAAGCGATG-3'
	reverse	5'-CAACGAGGCATTCTGGGATAG-3'
	probe	5'FAM-CCGGGTGAGGCACTACACTCTG-TAMRA3'

Supplementary Table 2.

Antibodies and staining reagents.

**Antibody**	**Clone**	**Conjugate**	**Catalog #**
CD45	30-F11	FITC	11-0451
CD45	30-F11	PE	12-0451
CD45	30-F11	PECy7	25-0451
CD45.2	104	FITC	11-0454
Ly-6G	1A8-Ly6g	PE	12-9668
Ly-6G	1A8-Ly6g	APC	17-9668
CD62L	MEL-14	PECy7	25-0621
CD115	AFS98	APC	17-1152
CD11b	M1/70	eFluor 450	48-0112
CD11b	M1/70	APC	561690
Ly-6C	HK1.4	APC-eFluor 780	47-5932
CD3	17A2	FITC	11-0032
CD19	eBio1D3 (1D3)	PECy7	25-0193
CD162	4RA10	PE	12-1621
CD11a	M17/4	eFluor 450	48-0111
CD49d	R1-2	PerCP-eFluor 710	46-0492
CD29	HMb1-1	APC-eFluor 780	47-0291
CXCR4	L276F12	APC	BZ-146507
CXCR2	IL-8RB	PerCP	FAB2164C
CD120a (TNFR1)	55R-286	PE	BZ-113003
Live/Dead Fixable Far Red			L34974

CXCR4 and CXCR2 antibodies were produced by Biolegend and R&D Systems, respectively, all other antibodies were manufactured by eBioscience. The LIVE/DEAD™ Fixable Far Red Dead Cell Stain Kit was manufactured by Life Technologies.

Supplementary Table 3.

Parameters of the fitted cosine curves shown in Figure 2B.

	spleen weight	
	AL	TRF
Mesor	70.64	60.67
Amplitude	7.45	8.18
Acrophase (ZT)	4.18	3.24
Cosinor fit, p-value	0.114	0.015

Supplementary Table 4.

Parameters of the fitted cosine curves shown in Figure 3B and D.

	per2		*reverbα*		*leptin*		serum leptin	
	AL	TRF	AL	TRF	AL	TRF	AL	TRF
Mesor	13.35	13.63	22.05	18.61	25.40	18.28	6627.71	4291.4
Amplitude	15.40	19.06	24.68	20.72	3.96	12.53	2495.47	3073.92
Acrophase (ZT)	14.23	13.84	8.57	9.45	16.97	17.66	21.96	19.79
Cosinor fit, p-value	0.071	0.165	0.000	0.011	0.771	0.018	0.072	0.003

Supplementary Table 5.

Parameters of the fitted cosine curves shown in Figure 5.

	Total WBC		B cell		T cell	
	AL	TRF	AL	TRF	AL	TRF
Mesor	17.00	17.54	6.13	6.67	4.29	4.35
Amplitude	8.59	9.99	3.99	4.26	1.60	1.99
Acrophase (ZT)	5.22	4.57	5.02	4.49	5.09	4.97
Cosinor fit, p-value	0.042	0.009	0.053	0.078	0.160	0.022
Increase in amplitude (%)		16		7		24

	Neutrophil		Monocyte		Ly6C^{high} Monocyte	
	AL	TRF	AL	TRF	AL	TRF
Mesor	2.54	2.77	1.65	1.51	1.17	1.01
Amplitude	1.01	1.58	0.51	0.98	0.38	0.79
Acrophase (ZT)	4.60	2.50	5.84	5.70	4.59	4.98
Cosinor fit, p-value	0.004	0.016	0.120	0.000	0.17	0.001
Increase in amplitude (%)		56		93		101

Supplementary Table 6.

Parameters of the fitted cosine curves shown in Supplementary Figure 7B.

	per2		*reverbα*	
	AL	TRF	AL	TRF
Mesor	2.38	2.29	2.48	2.65
Amplitude	0.51	1.09	1.05	0.83
Acrophase (ZT)	0.62	2.09	5.74	8.84
Cosinor fit, p-value	0.408	0.037	0.050	0.024

Supplementary Table 7.

Parameters of the cosine curves fitted on data shown in Supplementary Figure 7C, D, F.

	cxcl12		CXCL12		*tnfα*	
	AL	TRF	AL	TRF	AL	TRF
Mesor	6.75	5.85	2.02	2.04	4.18	4.13
Amplitude	2.45	3.32	0.30	0.39	0.71	1.59
Acrophase (ZT)	3.71	3.85	0.91	19.74	3.31	2.1
Cosinor fit, p-value	0.289	0.023	0.370	0.186	0.607	0.206
	nlrp3		*il1β*		*il18*	
	AL	TRF	AL	TRF	AL	TRF
Mesor	1.20	1.12	2.02	2.16	5.38	5.75
Amplitude	0.53	0.44	0.65	0.70	1.02	1.01
Acrophase (ZT)	1.26	2.29	22.46	21.77	4.81	6.45
Cosinor fit, p-value	0.197	0.098	0.081	0.030	0.033	0.035
